# Effects of tocolysis with nifedipine or atosiban on child outcome: follow‐up of the APOSTEL III trial

**DOI:** 10.1111/1471-0528.16186

**Published:** 2020-03-29

**Authors:** TMS van Winden, J Klumper, CE Kleinrouweler, MA Tichelaar, CA Naaktgeboren, TA Nijman, AL van Baar, AG van Wassenaer‐Leemhuis, TJ Roseboom, J van’t Hooft, C Roos, BW Mol, E Pajkrt, MA Oudijk

**Affiliations:** ^1^ Obstetrics, Amsterdam Reproduction and Development Research Institute Amsterdam UMC University of Amsterdam Amsterdam the Netherlands; ^2^ Julius Centre for Health Sciences and Primary Care University Medical Centre Utrecht Utrecht the Netherlands; ^3^ Department of Obstetrics and Gynaecology Leiden University Medical Centre Leiden the Netherlands; ^4^ Child and Adolescent Studies Utrecht University Utrecht the Netherlands; ^5^ Paediatrics, Amsterdam Reproduction and Development Research Institute Amsterdam UMC University of Amsterdam Amsterdam the Netherlands; ^6^ Department of Clinical Epidemiology, Biostatistics, and Bioinformatics Amsterdam UMC University of Amsterdam Amsterdam the Netherlands; ^7^ Department of Obstetrics and Gynaecology Monash University Clayton Victoria Australia

**Keywords:** Atosiban, behaviour, child, development, executive function, follow‐up, health, infant, neurodevelopment, nifedipine, preterm birth, preterm labour, tocolysis

## Abstract

**Objective:**

To compare the long‐term effects of tocolysis with nifedipine or atosiban on child outcome at age 2.5–5.5 years.

**Design:**

The APOSTEL III trial was a multicentre randomised controlled trial that compared tocolysis with nifedipine or atosiban in 503 women with threatened preterm birth. Neonatal outcomes did not differ between both treatment arms, except for a higher incidence of intubation in the atosiban group.

**Methods:**

Parents were asked to complete four questionnaires regarding neurodevelopment, executive function, behaviour problems and general health.

**Main outcome measures:**

The main long‐term outcome measure was a composite of abnormal development at the age of 2.5–5.5 years.

**Results:**

Of the 426 women eligible for follow‐up, 196 (46%) parents returned the questionnaires for 115 children in the nifedipine group and 110 children in the atosiban group. Abnormal development occurred in 32 children (30%) in the nifedipine group and in 38 children (38%) in the atosiban group (OR 0.74, 95% CI 0.41–1.34). The separate outcomes for neurodevelopment, executive function, behaviour, and general health showed no significant differences between the groups. Sensitivity analysis for all children of the APOSTEL III trial, including a comparison of deceased children, resulted in a higher rate of healthy survival in the nifedipine group (64 versus 54%), but there was no significant difference in the overall mortality rate (5.4 versus 2.7%). There were no significant subgroup effects.

**Conclusion:**

Outcomes on broad child neurodevelopment, executive function, behaviour and general health were comparable in both groups. Neither nifedipine nor atosiban can be considered as the preferred treatment for women with threatened preterm birth.

**Tweetable abstract:**

Nifedipine‐ and atosiban‐exposed children had comparable long‐term outcomes, including neurodevelopment, executive function and behaviour.

## Tweetable abstract

Nifedipine‐ and atosiban‐exposed children had comparable long‐term outcomes, including neurodevelopment, executive function, and behaviour.

## 1 Introduction

The effect of prenatal interventions, such as the use of tocolytics, on long‐term morbidity is largely unknown as follow‐up data, especially for older children, are scarce.^1^ Ultimately, the aim of treatment for threatened preterm birth should not be to increase gestational age at the time of birth, but to improve neonatal survival and healthy development.

It is therefore crucial to assess long‐term outcomes, because even if a randomised controlled trial (RCT) shows no difference between interventions on short‐term outcomes, the long‐term development of the children may still be affected. In the ORACLE II trial, in which antibiotic therapy was compared with placebo for threatened preterm labour with intact membranes, short‐term outcomes were similar between groups.^2^ At the 7‐year follow‐up, however, a significant increase in functional impairment and cerebral palsy was found in the group given antibiotics.^3^

The effect of two frequently used tocolytics, i.e. nifedipine and atosiban, on the long‐term health and development of children is largely unknown. Long‐term effects of nifedipine have only been investigated in three smaller studies.^4^, ^5^, ^6^ Only one retrospective cohort study on the long‐term effects of atosiban has been published, but this study was limited to autism spectrum disorders in children exposed to nifedipine alone or to nifedipine and atosiban in combination.^7^

The APOSTEL III study was a multicentre randomised trial that compared neonatal outcomes of tocolysis with nifedipine or atosiban in threatened preterm birth.^8^, ^9^ The primary outcome, a composite of neonatal morbidity and mortality, was comparable between the two arms (14% in the nifedipine group and 15% in the atosiban group, RR 0.91, 95% CI 0.61–1.37), although a non‐significant higher mortality rate was observed in the nifedipine group (5.4% in the nifedipine group and 2.4% in the atosiban group, RR 2.20, 95% CI 0.91–5.33).

The aim of this study was to determine the long‐term effects of tocolysis with nifedipine or atosiban during threatened preterm birth on neurodevelopment, executive function, behavioural problems and the general health of children.

## 2 Methods

### 2.1 Trial design and participants

The APOSTEL III study was a multicentre randomised controlled trial that analysed 503 women with threatened preterm birth and gestational age between 25^+0^ and 34^+0^ weeks of gestation, who were randomised to treatment with nifedipine (*n* = 248) or atosiban (*n* = 255). Both singleton and twin pregnancies were included.

The sample size of this follow‐up study was predefined by the number of participants of the APOSTEL III trial. In total, 503 mothers gave birth to 591 children (*n* = 297 treated with nifedipine and *n* = 294 treated with atosiban). There were 23 perinatal deaths: 16 in the nifedipine group and seven in the atosiban group.

In the design phase of the study, two patient organisations supported the study, and participated in the application for funding. Both Vereniging van Ouders van Couveusekinderen (VOC, a patient organisation for parents of children that were admitted to the neonatal intensive care unit) and Nederlandse Vereniging voor Ouders van Meerlingen (NVOM, the Dutch society of parents of multiples) were involved.

At the time that we applied for the funding of the follow‐up study, the methods were composed to the best of our knowledge at that moment. Before the actual analyses were performed, however, a number of changes were made in consultation with methodologists and experts. Those changes are marked and explained point by point in Appendix S1.

Five years after the start of the original trial, we asked all participants with a surviving child for written informed consent to send four questionnaires. Women with children older than 66 months were excluded, as the Ages & Stages Questionnaires^®^, Third Edition (ASQ‐3™) is not validated above that age. Data on mortality after finalisation of the RCT was gathered for all contactable participants.

### 2.2 Questionnaires

Four parent‐reported questionnaires were used, three of which are validated developmental questionnaires and one is aimed at gathering data about general health and healthcare use.

#### 2.2.1 ASQ‐3 – neurodevelopment

The ASQ‐3 questionnaire is used as a screening tool for delay in six domains of development.^10^ Scores were compared with a reference score file validated for the Dutch population.^11^ A questionnaire was marked abnormal if the score in at least one developmental field was ≥2 SD below the mean.^10^

#### 2.2.2 BRIEF‐P – executive function

The Behaviour Rating Inventory of Executive Function – Preschool (BRIEF‐P) is a standardised questionnaire to assess executive function, i.e. cognitive development and attention, in children aged between 2 and 5 years.^12^ The separate items describe different behavioural areas of executive functioning that together form the total score. Raw scores were converted into *T*‐scores and percentiles to correct for age and sex. Mean scores were compared with a norm score file validated for the Dutch population. A *T*‐score of 65 or higher (equivalent to 1.5 SD above the mean) on the scales, indices and total score was considered abnormal.^13^

#### 2.2.3 CBCL – behaviour

The Child Behaviour Check List (CBCL) questionnaire records behaviour and emotion in children aged between 1.5 and 5 years.^14^ The questions can be grouped into syndrome scales that inform on internalising and externalising behaviour. The syndrome scales form a total problem score. Scores were compared with the publisher’s reference file.^15^ *T*‐scores and percentiles were calculated. A *T*‐score of 64 or higher was considered abnormal.^14^

#### 2.2.4 General health

Data regarding medical history ___ i.e. hospital admissions; surgeries; visits to a general practitioner, medical specialist or developmental specialist; and past and present medication use ___ were collected.

### 2.3 Outcome measures

The main outcome was a composite of abnormal development at the age of 2.5–5.5 years. The proportion of children with abnormal scores on at least one of the development questionnaires and their subscales was compared between the nifedipine and atosiban groups. Secondary outcomes included general health outcomes, as described above.

### 2.4 Statistical analysis

For participants of follow‐up, we compared characteristics and outcomes between the nifedipine group and the atosiban group. The Mann–Whitney *U*‐test for continuous data, Fisher’s exact test for dichotomous data and χ^2^ test for categorical data were used, as appropriate.

For outcomes on the neonatal or child level, we accounted for interdependence between outcomes of babies from the same mother in multiple pregnancies.^16^, ^17^ We assessed binary outcomes with a generalised estimating equations (GEE) model for binomial data with an unstructured correlation matrix, considering the mother as a cluster variable. Odds ratios (ORs), 95% confidence intervals (95% CIs) and *P* values are reported.

Likewise, we evaluated continuous outcomes on the neonatal or child level with linear quantile mixed models with the mother as a grouping variable, resulting in a median difference with 95% CI.^18^ All long‐term child outcome analyses were adjusted for gestational age at birth.

We examined possible subgroup effects for women with and without intact membranes, singleton and multiple pregnancies, gestational age at delivery <32^+0^ versus ≥32^+0^ weeks of gestation and <35^+0^ versus ≥35^+0^ weeks of gestation. Subgroup effects were studied by including an interaction term between the subgrouping variable and treatment allocation in the regression model and were adjusted for gestational age at birth.

We performed a sensitivity analysis in which we included all children of the original APOSTEL III trial (*n* = 591). For all children who did not participate in the follow‐up, outcomes were estimated based on the results (rates of healthy survival and abnormal questionnaire score) of children who did participate, stratified for singleton and multiple pregnancies, and taking any deaths after the original trial into account.

We compared rates of healthy survival, i.e. all normal questionnaire scores and survival until the end of the follow‐up period, and all mortality between the nifedipine and atosiban group.

Data preparation and statistical analyses were performed using spss 25.0 (IBM Corp, Armonk, NY, USA) and r 3.5.1 (R Core Team, Vienna, Austria).

## 3 Results

### 3.1 Study population

**Figure 1:**
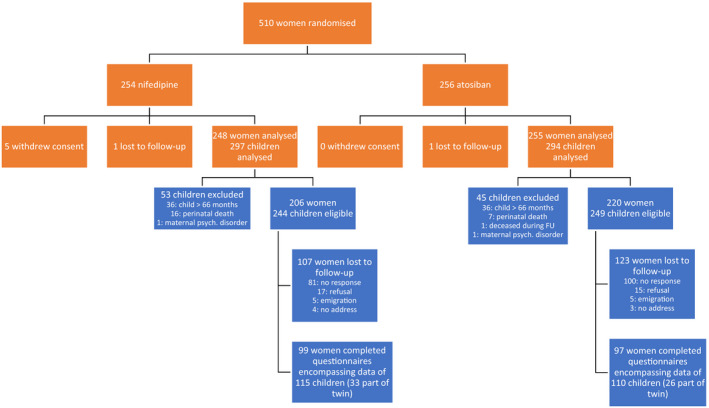
CONSORT flow diagram of the APOSTEL III trial and its follow‐up.^19^ 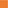, APOSTEL III RCT; 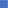, APOSTEL III Follow‐up

Out of 486 women and 568 surviving children, 426 women were eligible for follow‐up and 281 (66%) agreed to participate. Eventually, 196 (46%) of the eligible families returned the questionnaires, encompassing data for 115 infants randomised to the nifedipine group (51%) and 110 infants randomised to the atosiban group (49%), 33 (29%) and 26 (24%) of whom, respectively, were from a twin pregnancy (Figure 1). The median age of the children at the time of follow‐up was 53 months (interquartile range 46–57 months), with a slightly higher percentage of boys (58%). No differences were seen in these characteristics between both groups (Table S1).

Baseline maternal characteristics did not differ between both treatment arms in the participating group (Table 1). Short‐term neonatal outcomes for children up to a corrected age of 3 months in this follow‐up study showed a higher incidence of intubation in the atosiban group, whereas the median ventilation duration for nifedipine was non‐significantly longer (Table 2). Mothers in the participating group were older and were more often white, highly educated and nulliparous, compared with mothers in the non‐participating group (Table S2). There were no differences in short‐term neonatal outcomes when comparing children who did and did not participate in the follow‐up (Table S3).

**Table 1:** Baseline maternal characteristics according to treatment arm

	Nifedipine (*n* = 99)	Atosiban (*n* = 97)	*P*
**Age (years)**	31.1 (28.3–34.2)	30.6 (27.9–33.1)	0.34
**Body mass index (kg/m^2^)** ^a^	23.6 (21.5–26.0)	23.1 (21.2–25.7)	0.66
**White** ^b^	82 (89%)	82 (89%)	1.00
**Educational level** ^c^			
Primary school	1 (2.6%)	2 (5.4%)	0.51
Secondary school	0 (0.0%)	0 (0.0%)	
Lower professional education	2 (5.1%)	2 (5.4%)	
Medium professional education	13 (33%)	11 (30%)	
Higher professional education	18 (46%)	12 (32%)	
University	5 (13%)	10 (27%)	
**Nulliparous**	73 (74%)	75 (77%)	0.62
**History of preterm birth**	9 (9.1%)	8 (8.2%)	1.00
**Gestational age at randomisation**	30.6 (28.5–32.6)	30.6 (28.4–31.9)	0.47
**Multiple gestation**	17 (17%)	13 (13%)	0.55
**PPROM**	38 (38%)	31 (32%)	0.37
**Cervical length (mm)** ^d^	17.0 (9.0–22.2)	13.0 (7.0–17.5)	0.12
**Dilatation** ^e^	1.0 (1.0–2.0)	1.0 (0.0–2.5)	0.48

Outcome data are *n* (%) or median (IQR).

^a^ Nifedipine, *n* = 82; atosiban, *n* = 83.

^b^ Nifedipine, *n* = 92; atosiban, *n* = 92.

^c^ Nifedipine, *n* = 39; atosiban, *n* = 37.

^d^ Nifedipine, *n* = 64; atosiban, *n* = 55.

^e^ Nifedipine, *n* = 46; atosiban, *n* = 51.

**Table 2:** Short‐term neonatal outcomes of children participating in the follow‐up study^9^

	Nifedipine (*n* = 115)	Atosiban (*n* = 110)	*P*
**Gestational age at birth**	33^+0^ (30^+3^–35^+2^)	31^+6^ (29^+4^–34^+5^)	0.16
**Gestational age at birth**			
<28 weeks	10 (8.7%)	13 (11%)	0.44
28–32 weeks	38 (33%)	42 (38%)	
>32 weeks	67 (58%)	55 (50%)	
**Adverse perinatal composite outcome** ^a^	13 (11%)	15 (14%)	0.60
**Bronchopulmonary dysplasia**	4 (3.5%)	7 (6.4%)	0.33
**Culture‐proven sepsis**	11 (9.6%)	8 (7.3%)	0.54
**Intraventricular haemorrhage (grade ≥3)**	2 (1.7%)	0 (0.0%)	—
**Periventricular leukomalacia (grade ≥2)**	0 (0.0%)	1 (0.9%)	—
**Necrotising enterocolitis (stage ≥2)**	4 (3.5%)	1 (0.9%)	0.22
**NICU admission** ^b^	66 (57%)	71 (65%)	0.26
Length (days)^d^	14.5 (6.0–38.5)	18.0 (7.0–43.5)	0.75
**Intubation** ^c^	11 (9.6%)	24 (23%)	0.017
Length (days)^d^	6.0 (2.0–13.5)	2.5 (1.0–5.2)	0.082
**Any hospital admission**	103 (90%)	102 (93%)	0.41
**Days in hospital**	32.0 (18.5–54.0)	34.0 (21.0–62.0)	0.85
**Apnoea**	8 (7.0%)	13 (12%)	0.12
**Asphyxia**	0 (0.0%)	1 (0.9%)	—
**Proven meningitis**	2 (1.7%)	0 (0.0%)	—
**Birthweight (g)**	1920 (1553–2629)	1805 (1368–2419)	0.15

Outcome data are *n* (%) or median (IQR). Data are reported until the corrected age of 3 months.

^a^ Composed of perinatal in‐hospital mortality and the following perinatal morbidities: bronchopulmonary dysplasia, culture‐proven sepsis, intraventricular haemorrhage higher than grade 2, periventricular leukomalacia higher than grade 1 and necrotising enterocolitis higher than Bell’s stage 1.

^b^ Nifedipine, *n* = 115; atosiban, *n* = 109.

^c^ Nifedipine, *n* = 114; atosiban, *n* = 106.

^d^ Only reported for neonates who were admitted to the neonatal intensive care unit (NICU) or who underwent intubation.

### 3.2 Main outcome

#### 3.2.1 Developmental questionnaires

We received and included 225 ASQ‐3 questionnaires. We excluded any subscales that were filled in erroneously.^10^ In the BRIEF‐P analysis, 11 completed questionnaires were discarded for inconsistent and/or high negativity score (i.e. negative bias of the parent) and two questionnaires because of too many missing items. Ten CBCL questionnaires had too many missing items and were therefore excluded from the analysis. Developmental outcomes are shown in Table 3.

**Table 3:** Abnormal outcomes by treatment arm

	Domain	Nifedipine (*n* = 115)	Atosiban (*n* = 110)	OR	95% CI	*P*
		No. abnormal	No. abnormal			
Abnormal total score on any questionnaire	ASQ and/or BRIEF‐P and/or CBCL abnormal score	32/107 (30%)	38/100 (38%)	0.71	0.39–1.29	0.26
Neurodevelopment: ASQ‐3 abnormal^a^	Communication scale	9/113 (8.0%)	9/107 (8.4%)	0.79	0.28–2.17	0.64
	Gross motor scale	8/110 (7.3%)	7/106 (6.6%)	1.07	0.37–3.13	0.89
	Fine motor scale	18/112 (16%)	14/106 (13%)	1.46	0.65–3.29	0.36
	Problem solving scale	5/113 (4.4%)	13/107 (12%)	0.33	0.11–0.98	0.047
	Personal social scale	10/113 (8.8%)	10/107 (9.3%)	0.98	0.37–2.57	0.97
	Total neurodevelopmental delay	28/111 (25%)	30/107 (28%)	0.92	0.49–1.73	0.79
Executive function: BRIEF‐P abnormal^b^	Inhibitory self‐control index: inhibit and emotional control scales	9/108 (8.3%)	9/103 (8.7%)	0.94	0.36–2.47	0.90
	Flexibility index: shift and emotional control scales	10/108 (9.3%)	12/104 (12%)	0.73	0.30–1.77	0.48
	Emergent metacognition index: working memory and plan/organise scale	6/108 (5.6%)	10/103 (9.7%)	0.66	0.23–1.94	0.45
	Total executive function disorders	9/108 (8.3%)	10/102 (9.8%)	0.79	0.31–2.01	0.62
Behaviour: CBCL abnormal^c^	Internalising scale	10/111 (9.0%)	7/104 (6.7%)	1.32	0.48–3.62	0.59
	Externalising scale	9/111 (8.1%)	5/104 (4.8%)	1.67	0.55–5.08	0.36
	Total behavioural problems	10/111 (9.0%)	7/104 (6.7%)	1.31	0.46–3.74	0.62

According to the respective manuals, the cut‐off values for defining an abnormal score are:

^a^ At least one developmental field scoring ≥2 SD below the mean.

^b^ *T*‐score of 65 or higher.

^c^ *T*‐score of 64 or higher.

Neurodevelopmental delay (ASQ‐3) did not differ between the nifedipine and atosiban groups (25 versus 28% abnormal score, OR 0.92, 95% CI 0.50–1.71).

Executive function disorders (BRIEF‐P) were also similar in both groups (8.3 versus 9.8% abnormal score, OR 0.81, 95% CI 0.32–2.04). Behavioural problems (CBCL) were also comparable between the nifedipine and atosiban groups (9.0 versus 6.7% abnormal score, OR 1.32, 95% CI 0.47–3.72). Summarising the outcomes of the ASQ‐3, BRIEF‐P and CBCL questionnaires, there were no significant differences between the nifedipine and atosiban exposed groups (30 versus 38% with any abnormal questionnaire score, OR 0.74, 95% CI 0.41–1.34). Only for the ASQ‐3 problem‐solving scale was there a significantly lower incidence of abnormal scores in the nifedipine group (4.4 versus 12.0%, OR 0.34, 95% CI 0.12–0.99).

#### 3.2.2 General health questionnaire

No differences in general health were found between the nifedipine and atosiban groups with respect to hospital admissions, surgery, specialist visits (divided into general practitioner, developmental specialist and medical specialist) and medication use. Given that no differences were found for different subtypes (e.g. antibiotics or anti‐epileptics), we only reported on total medication use (Table 4).

**Table 4:** General health outcomes

	Nifedipine (*n* = 115)	Atosiban (*n* = 110)	OR	95% CI	*P*
**Hospital admissions (any)**	61/108 (57%)	53/108 (49%)	1.45	0.84–2.51	0.18
**Hospital admissions (≥3)**	14/108 (13%)	6/108 (5.6%)	3.18	1.05–9.66	0.041
**Surgery (any)**	38/110 (35%)	38/109 (35%)	1.03	0.58–1.84	0.92
**Surgery (≥3)**	8/110 (7.3%)	6/109 (5.6%)	1.54	0.48–4.90	0.47
**Any specialist visits**	91/112 (81%)	84/108 (78%)	1.47	0.72–3.00	0.29
General practitioner	65/112 (58%)	64/107 (60%)	0.98	0.55–1.76	0.96
Developmental specialist	55/110 (50 %)	48/107 (50%)	1.41	0.78–2.55	0.26
Medical specialist	79/112 (71%)	70/108 (65%)	1.43	0.76–2.68	0.27
**Medication use (ever)**	84/110 (76%)	75/109 (69%)	1.55	0.80–3.00	0.19
**Medication use (current)**	26/110 (24%)	25/109 (23%)	1.06	0.55–2.04	0.85

### 3.3 Subgroup analysis

There were no significant interactions (*P* < 0.05) between treatment allocation and subgroups of women with and without intact membranes, singleton and multiple pregnancies, gestational age at delivery <32^+0^ versus ≥32^+0^ weeks of gestation and <35^+0^ versus ≥35^+0^ weeks of gestation (Table S4).

### 3.4 Sensitivity analysis

A total of 24 children died during the study: 23 in the perinatal period and one during follow‐up. In a sensitivity analysis, the overall neonatal and childhood mortality from randomisation until follow‐up was 16/297 in the nifedipine group and 8/294 in the atosiban group (5.4 versus 2.7%, OR 2.06, 95% CI 0.86–4.95). There were 190/297 healthy survivors in the nifedipine group versus 159/294 healthy survivors in the atosiban group (64 versus 54%, OR 1.50, 95% CI 1.06–2.12), and there were 91/297 children with any abnormal questionnaire score in the nifedipine group versus 127/294 children in the atosiban group (31 versus 43%, OR 0.58, 95% CI 0.41–0.83).

## 4 Discussion

### 4.1 Main findings

In this long‐term follow‐up study, we found comparable outcomes in children exposed in utero to nifedipine or atosiban in the composite broad developmental scores, as well as in individual neurodevelopmental, executive functional, behavioural and health outcomes. We did find a significant difference in the ASQ‐3 problem‐solving scale in favour of nifedipine. No additional interactions could be found between treatment allocation and subgroups with respect to intact or ruptured membranes, singleton and multiple pregnancies and gestational age at delivery.

### 4.2 Strengths and limitations

There are several strengths of this study. First, this is the first published follow‐up study of an RCT describing the long‐term effects of atosiban on children’s development. This study contributes to the available knowledge about the long‐term effects of two widely used tocolytics.

Second, responses to the questionnaires were scrutinised and were not used if there were any doubts about the veracity of the responses.

Third, the response rate of 46% in our study is reasonable, considering that follow‐up studies in the same field yielded similar rates,^6^, ^20^, ^21^ and the extensive effort leading to this result.

Several limitations require comment. First, the sample size was limited by the number of participants of the APOSTEL III trial willing to participate in the follow‐up. To investigate the representativeness of the sample, baseline maternal and short‐term neonatal outcomes (i.e. up until discharge) were compared between participants and non‐participants of the follow‐up study. We found small differences in maternal baseline characteristics and short‐term neonatal outcomes between both groups. This is consistent with the phenomenon that participants of follow‐up research are generally older, more often white, higher educated and nulliparous than non‐participants.^22^

Second, because of the non‐significantly higher mortality rate in the nifedipine group of the APOSTEL III trial, there is a risk of bias because deceased and very disabled infants are not able to participate in the follow‐up. We investigated this in a sensitivity analysis encompassing all children of the original trial, including cases of perinatal and child mortality. This showed no significant differences in overall mortality. For surviving children, the rate of healthy survival was significantly higher in the nifedipine group. This can only be regarded as an exploratory analysis, however, and the result should be interpreted with caution. Outcomes of children who did not participate could only be estimated based on those of children who did participate in the follow‐up, thereby assuming that these groups are similar, whereas it is more likely that they are not. When data are not missing at random, as is probably the case in this study according to the follow‐up baseline characteristics, all imputation techniques may lead to inappropriate conclusions.

Third, the choice of measuring the outcome by using questionnaires requires comment. Obviously, objective data from a professional observer in addition to the use of parent‐reported questionnaire data would be preferable, although with large numbers of children this is difficult and costly. Bringing children in for testing would have no doubt further dwindled the numbers recruited to the study groups. The questionnaires that we used are regarded as validated screening tools for broad developmental problems. Moreover, our aim was not to make exact clinical diagnoses but purely to demonstrate a potential difference between the groups.

### 4.3 Interpretation (in light of other evidence)

Long‐term follow‐up of children exposed to tocolytics is scarce. Only 16% of the large perinatal RCTs, including tocolysis studies, report a follow‐up of children, whereas the development of the child is, besides child mortality, the most important outcome.^23^

We believe that for making a proper assessment of the superiority of either tocolytic, one should consider both short‐term and long‐term outcomes, and stress that long‐term follow‐up should become standard practice in all obstetric intervention trials.

The previous APOSTEL II follow‐up study compared the long‐term outcomes of maintenance treatment with nifedipine versus placebo, using the ASQ‐3 questionnaire.^6^ Overall, nifedipine‐exposed children scored more poorly on the fine‐motor scale, but did better on the problem‐solving scale. Two other studies compared the long‐term outcome of children exposed to ritodrine and nifedipine. No differences between the groups were found.^4^, ^5^ This study, therefore, contributes important information on the broad development of children exposed to tocolytics.

Clinically, preterm birth is strongly associated with long‐term developmental problems. In our study, the nifedipine group had both a non‐significant higher healthy survival and a higher mortality rate. In vitro studies have demonstrated a potential neuroprotective effect of nifedipine, which could be a pathophysiological explanation for the better scores on the problem‐solving scale in the nifedipine group.^24^ Atosiban could have had a direct effect on the fetal brain, although only a small portion of the peptide reaches the child’s brain after placental transfer.^25^

There were small differences in short‐term neonatal outcomes among follow‐up participants, where atosiban‐exposed children more often required intubation and nifedipine‐exposed children had a non‐significantly longer duration of intubation.

Based on our study, there seems to be a trade‐off in the outcome. This can be taken into consideration when counselling a patient, although there is no compelling evidence to favour one tocolytic over the other.

## 5 Conclusion

The APOSTEL III RCT found no differences in adverse perinatal outcomes in infants exposed to nifedipine or atosiban, and neither did this follow‐up study in long‐term outcomes. Based on this evidence, there is no preference for either nifedipine or atosiban in threatened preterm birth.

### 5.1 Disclosure of interests

BWM reports consultancy for ObsEva, Merck and Guerbet. BWM is supported by a National Health and Medical Research Council (NHMRC) Practitioner Fellowship (GNT1082548). All other authors report no conflicts of interest. Completed disclosure of interests form available to view online as supporting information.

### 5.2 Contribution to authorship

TMSW, CEK and CAN had full access to all of the data in the study and take responsibility for the integrity of the data and the accuracy of the data analysis. Study concept and design: TMSW, MAO and BWM. Acquisition, analysis, or interpretation of the data: TMSW, MAO, CEK, MAT, CR, CAN, ALB, AGW‐L, TJR, JH, BWM and EP. Drafting the manuscript: TMSW, MAO and CR. Critical revision of the manuscript for important intellectual content: JK, CEK, MT, CAN, TAN, ALB, AGW‐L, TJR, JH, BWM and EP. Statistical analysis: TMSW, CEK, MAT and CAN. Study supervision: MAO, CR and EP.

### 5.3 Details of ethics approval

The study was approved by the Research Ethics Committee of the Amsterdam UMC (ref. HREC AMC W15_039, 11 February 2015), with the note that the Medical Research Involving Human Subjects Act does not apply to this study as no negative consequences for mother or child can be expected by participating. Parents of all participating children provided written informed consent.

### 5.4 Funding

This study was funded by ZonMw, the Netherlands Organisation for Health Research and Development ‘Healthcare Rational Medicine’ programme, project number 836041012. The original APOSTEL III study was also financially supported by ZonMw under project number 836011005, NTR2947. ZonMw had no role in the study design, collection, analysis and interpretation of the data, writing of the report and decision to submit the article for publication.

### 5.5 Acknowledgements

We thank ZonMw, the Netherlands Organisation for Health Research and Development, for enabling and supporting clinical research. We thank the research nurses, midwives and administrative assistants of our consortium, and the nurses, midwives, residents and gynaecologists of the participating centres for their help with participant recruitment and data collection. We thank Dr C. van de Beek for sharing her experience regarding long‐term follow‐up in obstetric studies. We offer special thanks to Dr Jannet J.H. Bakker for her willingness to assist with the early phases of the study.

## Supporting information


**Table S1.** Characteristics of children participating in the follow‐up study.

**Table S2.** Baseline maternal characteristics of participants included in the follow‐up versus those not included in the follow‐up.

**Table S3.** Short‐term neonatal outcomes of participants included in the follow‐up versus those not included in the follow‐up.

**Table S4.** Subgroup analyses.

**Appendix S1.** Protocol showing changes made in consultation with methodologists or expert co‐authors before the actual analyses were performed.

 > Click here for additional data file.

 > Click here for additional data file.

 > Click here for additional data file.

 > Click here for additional data file.

 > Click here for additional data file.

 > Click here for additional data file.

 > Click here for additional data file.

 > Click here for additional data file.

 > Click here for additional data file.

 > Click here for additional data file.

 > Click here for additional data file.

 > Click here for additional data file.

 > Click here for additional data file.

 > Click here for additional data file.
