# Supplementary material for: Effects of tocolysis with nifedipine or atosiban on child outcome: follow‐up of the APOSTEL III trial
Source: BJOG. 2020 Mar 29;127(9):1129–37. doi: 10.1111/1471-0528.16186 (PMC7384124; doi:10.1111/1471-0528.16186)
Supplement: Supplementary file 1 — Table S1. Characteristics of children participating in the follow‐up study. [file BJO-127-1129-s001.pdf]

Table S1. Characteristics of children participating in follow-up study

	**Nifedipine (n=115)**	**Atosiban (n=110)**	**P-value**
Age (months), median (IQR)	51.1 (45.9 - 56.7)	53.4 (46.4 - 58.3)	0.14
30 - 36 months	3 (2.6%)	2 (1.8%)	
36 - 42 months	11 (9.6%)	3 (2.7%)	
42 - 48 months	22 (19%)	28 (26%)	
48 - 54 months	35 (30%)	26 (24%)	
54 - 60 months	0 (0.0%)	0 (0.0%)	
60 - 66 months	44 (38%)	51 (46%)	
Sex (boys), N (%)	71 (62%)	59 (54%)	0.22
Twins*, N (%)	33 (29%)	26 (24%)	0.30

*: including 1 child born from a twin pregnancy in which the sibling did not survive.
