# Supplementary material for: Effects of tocolysis with nifedipine or atosiban on child outcome: follow‐up of the APOSTEL III trial
Source: BJOG. 2020 Mar 29;127(9):1129–37. doi: 10.1111/1471-0528.16186 (PMC7384124; doi:10.1111/1471-0528.16186)
Supplement: Supplementary file 2 — Table S2. Baseline maternal characteristics of participants included in the follow‐up versus those not included in the follow‐up. [file BJO-127-1129-s002.pdf]

Table S2. Baseline maternal characteristics of participants included in follow up vs not in follow up

	Included in follow up (n=196)	Not in follow up (n=307)	P-value
Age (years), median (IQR)	30.8 (28.2 - 33.9)	29.9 (25.6 - 33.3)	0.032
BMI, median (IQR)*	23.4 (21.2 - 25.8)	22.4 (20.4 - 25.4)	0.053
Caucasian†	164 (89%)	199 (76%)	0.0009
Educational level‡			0.028
Primary school	3 (3.9%)	3 (2.8%)	
Secondary school	0 (0.0%)	6 (5.6%)	
Lower professional education	4 (5.3%)	11 (10%)	
Medium professional education	24 (32%)	48 (44%)	
Higher professional education	30 (40%)	25 (23%)	
University	15 (19.7%)	15 (14%)	
Nulliparous§	148 (76%)	181 (59%)	0.0002
History of preterm birth||	17 (8.7%)	46 (15%)	0.048
Gestational age at randomisation, median (IQR)	30.6 (28.4 - 32.3)	30.9 (28.9 - 33.0)	0.091
Multiple gestation	31 (16%)	57 (18%)	0.47
PPROM	69 (35%)	104 (34%)	0.77
Cervical length¶	14.0 (8.0-22.0)	14.0 (9.0-23.0)	0.81
Dilatation¶	1.0 (1.0-2.0)	1.0 (1.0-2.0)	0.35

Data are presented as n (%) or median (IQR). *: in FU n=165 and not in FU n=240, †: in FU n=184 and not in FU n=261, ‡: in FU n=76 and not in FU n=108, §: in FU n=196 and not in FU n=306, ||: in FU n=196 and not in FU n=305, V†: in FU n=119 and not in FU n=192, ¶: in FU n=97 and not in FU n=135.
