# Supplementary material for: Effects of tocolysis with nifedipine or atosiban on child outcome: follow‐up of the APOSTEL III trial
Source: BJOG. 2020 Mar 29;127(9):1129–37. doi: 10.1111/1471-0528.16186 (PMC7384124; doi:10.1111/1471-0528.16186)
Supplement: Supplementary file 3 — Table S3. Short‐term neonatal outcomes of participants included in the follow‐up versus those not included in the follow‐up. [file BJO-127-1129-s003.pdf]

Table S3. Short-term neonatal outcomes of participants included in follow up vs not in follow up

	Included in follow up (n=225)	Not in follow up (n=366)	P-value
Adverse perinatal composite outcome*	28 (12%)	59 (16%)	0.26
Bronchopulmonary dysplasia†	11 (4.9%)	21 (5.8%)	0.74
Culture-proven sepsis†	19 (8.4%)	31 (8.5%)	0.84
Intraventricular haemorrhage (grade ≥3)†	2 (0.9%)	5 (1.4%)	0.60
Periventricular leukomalacia (grade >2)†	1 (0.4%)	2 (0.5%)	0.87
Necrotising enterocolitis (stage >2)†	5 (2.2%)	6 (1.6%)	0.62
NICU admission	137 (61%)	201 (55%)	0.18
Length of NICU admission (days)‡	15 (5.5-38)	17 (7-40)	0.49
Intubation§	35 (17%)	62 (19%)	0.49
Time on intubation (days)||	4.0 (1.0-7.5)	3.0 (1.0-8.0)	0.64
Any hospital admission	209 (93%)	330 (90%)	0.26
Days in hospital until 3 months corrected age¶	32 (18-55)	26 (10-47)	0.15
Apnoea†	21 (9.3%)	24 (6.6%)	0.31
Asphyxia†	1 (0.4%)	3 (0.8%)	0.59
Proven meningitis†	2 (0.9%)	5 (1.4%)	0.60
Birthweight (g)#	1845 (1457-2540)	2000 (1525-2650)	0.21

Outcome data are n (%) or median (IQR).
*: composed of perinatal in-hospital mortality and the following perinatal morbidities: bronchopulmonary dysplasia, culture-proven sepsis, intraventricular haemorrhage > grade 2, periventricular leukomalacia > grade 1, and necrotising enterocolitis > Bell's stage 1.
†: in FU n=225 and not in FU n=364, ‡: in FU n=135 and not in FU n=194, §: in FU n=203 and not in FU n=319, ||: in FU n=35 and not in FU n=61, ¶: in FU n=207 and not in FU n=324, #: only live births. in FU n=225 and not in FU n=343.
