# Supplementary material for: Effects of tocolysis with nifedipine or atosiban on child outcome: follow‐up of the APOSTEL III trial
Source: BJOG. 2020 Mar 29;127(9):1129–37. doi: 10.1111/1471-0528.16186 (PMC7384124; doi:10.1111/1471-0528.16186)
Supplement: Supplementary file 4 — Table S4. Subgroup analyses. [file BJO-127-1129-s004.pdf]

Table S4. Subgroup analyses

Outcome	Subgroups	Nifedipine (n=115)	Atosiban (n=110)	p-value interaction term
**PPROM vs no PPROM**				
**Composite outcome:**	PPROM (n=80)	8/40 (20%)	13/33 (39%)	0.15
**ASQ and/or BRIEF-P and/or CBCL abnormal**	No PPROM (n=145)	24/67 (36%)	25/67 (37%)	
**ASQ-3:**	PPROM (n=80)	8/42 (19%)	11/36 (31%)	0.21
**Total developmental delay**	No PPROM (n=145)	20/69 (29%)	19/71 (27%)	
**BRIEF-P:**	PPROM (n=80)	2/40 (5.0%)	3/32 (9.4%)	0.50
**Total executive function disorders**	No PPROM (n=145)	7/68 (10%)	7/70 (10%)	
**CBCL:**	PPROM (n=80)	2/43 (4.7%)	1/35 (2.9%)	0.89
**Total behavioural problems**	No PPROM (n=145)	8/68 (12%)	6/69 (8.7%)	
**Singleton vs multiple pregnancy**				
**Composite outcome:**	Singleton pregnancy (n=166)	26/77 (34%)	32/77 (42%)	0.96
**ASQ and/or BRIEF-P and/or CBCL abnormal**	Multiple pregnancy (n=59)	6/30 (20%)	6/23 (26%)	
**ASQ-3:**	Singleton pregnancy (n=166)	24/78 (31%)	26/83 (31 %)	0.70
**Total developmental delay**	Multiple pregnancy (n=59)	4/33 (12%)	4/24 (17%)	
**BRIEF-P:**	Singleton pregnancy (n=166)	8/78 (10%)	6/76 (7.9%)	0.12
**Total executive function disorders**	Multiple pregnancy (n=59)	1/30 (3.3%)	4/26 (15%)	
**CBCL:**	Singleton pregnancy (n=166)	7/80 (8.8%)	6/79 (7.6%)	0.57
**Total behavioural problems**	Multiple pregnancy (n=59)	3/31 (9.7%)	1/25 (4.0%)	
**Gestational age at delivery <32 vs ≥32 weeks**				
**Composite outcome:**	GA at delivery < 32 weeks (n=103)	16/46 (35%)	18/48 (38%)	0.56
**ASQ and/or BRIEF-P and/or CBCL abnormal**	GA at delivery ≥ 32 weeks (n=122)	16/61 (26%)	20/52 (39%)	
**ASQ-3:**	GA at delivery < 32 weeks (n=103)	15/47 (32%)	13/53 (25%)	0.18
**Total developmental delay**	GA at delivery ≥ 32 weeks (n=122)	13/64 (20%)	17/54 (32%)	
**BRIEF-P:**	GA at delivery < 32 weeks (n=103)	3/44 (6.8%)	5/50 (10%)	0.68
**Total executive function disorders**	GA at delivery ≥ 32 weeks (n=122)	6/64 (9.4%)	5/52 (9.6%)	
**CBCL:**	GA at delivery < 32 weeks (n=103)	5/48 (10%)	2/52 (3.8%)	0.36
**Total behavioural problems**	GA at delivery ≥ 32 weeks (n=122)	5/63 (7.9%)	5/52 (9.6%)	
**Gestational age at delivery <35 vs ≥35 weeks**				
**Composite outcome:**	GA at delivery < 35 weeks (n=166)	27/77 (35%)	29/74 (39%)	0.27
**ASQ and/or BRIEF-P and/or CBCL abnormal**	GA at delivery ≥ 35 weeks (n=59)	5/30 (17%)	9/26 (35%)	
**ASQ-3:**	GA at delivery < 35 weeks (n=166)	24/81 (30%)	24/80 (30%)	0.44
**Total developmental delay**	GA at delivery ≥ 35 weeks (n=59)	4/30 (13%)	6/27 (22%)	
**BRIEF-P:**	GA at delivery < 35 weeks (n=166)	7/77 (9.1%)	8/76 (11%)	0.99
**Total executive function disorders**	GA at delivery ≥ 35 weeks (n=59)	2/31 (6.5%)	2/26 (7.7%)	
**CBCL:**	GA at delivery < 35 weeks (n=166)	9/81 (11%)	5/79 (6.3%)	0.28
**Total behavioural problems**	GA at delivery ≥ 35 weeks (n=59)	1/30 (3.3%)	2/25 (8.0%)	

The interaction term (p-value) indicates whether there is an interaction between treatment allocation and outcome for subgroups, i.e. whether the effect of treatment allocation on outcome is meaningfully different for the different subgroups.
