# Supplementary material for: Effects of tocolysis with nifedipine or atosiban on child outcome: follow‐up of the APOSTEL III trial
Source: BJOG. 2020 Mar 29;127(9):1129–37. doi: 10.1111/1471-0528.16186 (PMC7384124; doi:10.1111/1471-0528.16186)
Supplement: Supplementary file 5 — Appendix S1. Protocol showing changes made in consultation with methodologists or expert co‐authors before the actual analyses were performed. [file BJO-127-1129-s005.pdf]

Appendix S1. Protocol showing changes made in consultation with methodologists or expert co-authors before the actual analyses were performed.

MEASUREMENTS: All parents of live born children who participated in the APOSTEL III trial will be requested to fill out the ASQ, BRIEF-P and CBCL-questionnaires. Favorably the questionnaires are filled out at 2.5- 3.5 years of age, corrected for prematurity. When not reached at the preferable age, questionnaires appropriate for the age will be used.
The Ages & Stages Questionnaire (ASQ) will be used to investigate (neuro)developmental outcomes. The ASQ is a screening tool used to identify developmental problems. Parents have to answer questions regarding 5 areas of development of their child. These areas include communication, gross motor, fine motor, problem solving and personal-social. Mean scores will be calculated for each area, scores ≥1 SD below the expected score is considered abnormal. The ASQ has proven to be a valuable screening instrument for detecting developmental delay in ex-premature infants (20), and the instrument is not influenced by the socio-economic status of the parents (21). Although parents with language problems might have difficulties with completing the questionnaires.
The Child Behavioral Checklist (CBCL) will be used to investigate behavioral outcomes (22). This list contains 99 questions regarding behavioral and emotional difficulties, observed by parents. Problems known to occur simultaneously are clustered in the following narrow-band problem scales: emotional reactive, anxious/depressed, physical complaints, withdrawal, sleeping difficulties, attention problems and aggressive behavior. The first four scales form the broad-band scale internalizing problems and the latter three externalizing problems. All problem scales together form the total problem scale. For each scale a standardized t score is calculated. A score >97th percentile indicates (serious) behavior problems.
Furthermore we will collect data on height and weight from birth until the age of 2.5 to 3.5 years, using data collected in the individual growth book of each child (known in Dutch as 'het groeiboekje') as well as on children's health (diagnoses, treatments, and hospital admissions) admittance.

OUTCOMES:
Primary outcome is poor (neuro)developmental of behavioral outcome. Poor outcome is defined as a score of -1 SD using 1 SD for the total ASQ-3 score and 2 SD on any subscale as cut-off points and a score >97th percentile for the CBCL. Secondary outcomes will be growth of the children and their general health (diagnoses, treatments and hospital admissions).

SAMPLE SIZE:
The size of the study is predefined by the number of participants of the APOSTEL-III trial: 588 children were born; 10% of the infants were born at <28 weeks of gestation, 11% at 28-30 weeks; 21% at 30-32 weeks; and 58% at >32 weeks of gestation. There were 22 perinatal deaths, therefore, 566 children can be approached.

STATISTICAL ANALYSIS:
Demographic maternal and neonatal characteristics will be compared between the groups. The amount of children with abnormal scores for ASQ and the CBCL will be compared in the group exposed to nifedipine versus atosiban, using the χ -test. T-tests will be used to compare mean scores in the groups per area in both questionnaires. To identify factors with

Commented [TvW1]: The BRIEF-P (Behaviour Rating Inventory of Executive Function – Preschool) is a standardised questionnaire to assess executive function, i.e. cognitive development and attention – an area that is highly important when assessing broad child neurodevelopment. By including the BRIEF-P questionnaire, we aimed to close this gap.

Commented [TvW2]: We chose not to correct for prematurity. Firstly, because most of the questionnaires (ASQ and BRIEF-P) are already adjusted for prematurity. A second reason is that the age of the children included in the study was well above 24 months (median 52 months). In literature, the general consensus is to correct below this age. Above 24 months, there is no evidence to calculate . It makes more sense to adjust for gestational age at birth, which we did both in the main analyses as well as in the subgroup analyses.

Commented [TvW3]: Instead, we chose to follow the questionnaire's manual (*a questionnaire was marked abnormal if the score in at least one developmental field was ≥2 SD under the mean*) in order to obtain the most reliable and validated results.

Commented [TvW4]: Just as for the ASQ questionnaire, we stuck with the questionnaire's manual: *A T-score of 64 or higher was considered abnormal.*

Commented [TvW5]: **Huidige stuk: The main outcome was a composite of abnormal development at the age of 2.5-5.5 years. The proportion of children with abnormal scores on at least one of the development questionnaires and their subscales was compared between the nifedipine and atosiban group. Secondary outcomes included general health outcomes as described above.**
→ wat mij betreft zo laten, hoewel iets anders gedefinieerd is het niet wezenlijk anders dan wat er in het protocol beschreven staat. Eens?

Commented [TvW6]: In line with changes above

impact on the ASQ and CBCL univariate analyses will be performed using χ -test, comparing children with an abnormal test results to those with normal scores. Items with a p- value <0.10 will be entered in a logistical regression model, either as continuous or as categorical variables, to assess the joint influence on the outcome of the ASQ and CBCL test.
